# Novel Insights for Systemic Inflammation in Sepsis and Hemorrhage

**DOI:** 10.1155/2010/642462

**Published:** 2010-06-08

**Authors:** Bolin Cai, Edwin A. Deitch, Luis Ulloa

**Affiliations:** Laboratory of Anti-Inflammatory Signaling and Surgical Immunology, Department of Surgery, UMDNJ-New Jersey Medical School, Medical Science Building F-673, 185 South Orange Avenue, P.O. Box 1709, Newark, NJ 07103, USA

## Abstract

The inflammatory responses in sepsis and hemorrhage remain a major cause of death. Clinically, it is generally accepted that shock in sepsis or hemorrhage differs in its mechanisms. However, the recognition of inflammatory cytokines as a common lethal pathway has become consent. Proinflammatory cytokines such as tumor necrosis factor (TNF) or high-mobility group box1 (HMGB1) are fanatically released and cause lethal multiorgan dysfunction. Inhibition of these cytokines can prevent the inflammatory responses and organ damage. In seeking potential anti-inflammatory strategies, we reported that ethyl pyruvate and alpha7 nicotinic acetylcholine receptor (alpha7nAChR) agonists effectively restrained cytokine production to provide therapeutic benefits in both experimental sepsis and hemorrhage. Here, we review the inflammatory responses and the anti-inflammatory strategies in experimental models of sepsis and hemorrhage, as they may have a consistent inflammatory pathway in spite of their different pathophysiological processes.

## 1. Introduction

Bacterial toxins can cause respiratory and cardiac failure or even death within the first 24–48 hours after infection. The body's responses to foreign invasion can in some cases, depending on the amount of insult, initiate an immunologic protection to eliminate the insult. A fanatical production of proinflammatory cytokines such as TNF produced during endotoxemia may cause organ injury or eventual death [1, 2], even though TNF may be required in healing the damaged tissues caused by infection or injury in the early stage or mild inflammatory response. In mounting reports and the experiments of sepsis and other stresses such as trauma and acute hemorrhage carried out by us recently, the deleterious inflammatory responses were obviously evidenced to present a variety of signs and symptoms of cardiovascular collapse and multiorgan dysfunctions, which can be prevented by inhibition TNF [3, 4]. In seeking anti-inflammatory supplements to restrain TNF production, we found the ethyl pyruvate and alpha7nAChR agonists effectively inhibit TNF production in sepsis and hemorrhage [5, 6]. 

In addition to TNF, high mobility group B protein-1 (HMGB1) is a characteristic “late” factor contributing to endothelial leakage, vascular collapse, acute lung injury, heart failure, or even sudden cardiac death. HMGB1 is a constitutive intracellular protein that is released into the extracellular milieu from damaging cells in response to the inflammation during hemorrhage or sepsis. Following acute inflammation, the immune system is able to recognize the HMGB1 as a hazard molecule. In looking for anti-inflammatory therapeutic strategies, we have also found ethyl pyruvate and alpha7nAcChR agonist choline posses a potential inhibition of HMGB1 leading to improvement of animal survival after hemorrhage or sepsis [5–7]. Here, we review the new insights on the mentions. 

## 2. Systemic Inflammation in Sepsis

Sepsis is one of the most common causes of death in hospitalized patients accounting for 9.3% of overall deaths in the United States annually. Sepsis affects over 18 million people worldwide and it is expected 1% increase in incidence per year [1, 2]. Sepsis is characterized by bacterial infection with at least clinical manifestations of the following: abnormalities of body temperature (hypothermia or hyperthermia), heart rate (tachycardia), respiratory rate (tachypnea), and white blood cell count (leukocytopenia or leukocytosis). Two clinical syndromes have been associated with sepsis. “Septic shock” is highly lethal syndrome of cardiovascular shock that kills within 24–48 hours since onset. Shock is invariably accompanied by ischemic necrosis and cardiovascular collapse. The second syndrome is a more protracted condition called “severe sepsis”, which refers to an association with organ dysfunction, such as hypoxemia, oliguria, lactic acidosis, and elevated liver enzymes or altered cerebral function [3, 4]. This less acute syndrome kills more slowly, progressing over 7–14 days with a mortality rate of 30–70%. In some cases, these two syndromes represent two different stages in the progression of sepsis. If patients survive to the acute episode of septic shock, they may develop a state of severe sepsis characterized by progressive organ damage in the liver, kidneys, and lungs [8]. However, these two syndromes are, in fact, independent disorders, and not all patients with septic shock develop severe sepsis, and vice-versa. 

Despite the recent advances in antibiotics and critical care, severe sepsis remains a major cause of death, in part because antibiotics cannot control systemic inflammation and severe sepsis is not exclusively produced by infections. Trauma, ischemia, and severe injury also contribute to the pathogenesis of the syndrome, which is characterized by an overwhelming production of inflammatory cytokines including TNF, interleukin (IL)-1, and HMGB1. These cytokines trigger beneficial inflammatory responses to confine the infection and tissue damage. However, the excessive production of these cytokines can produce inflammatory responses, being even more death-defying than the original trauma or infection [9–11]. This concept is especially notable in severe sepsis, where the excessive production of inflammatory cytokines causes capillary leakage, tissue injury, and lethal multiple organ failure. Early cytokines like TNF and IL-1 peak within the first hours after the infection, then circulatory levels revert to near baseline by 3-4 hours [9, 11]. This characteristic kinetic of “early” cytokines provides a narrow therapeutic window for clinical intervention. 

“Late” mediators of sepsis are those cytokines that are produced in the later stages of the pathogenesis. The HMGB1 is one of the delayed cytokines secreted by macrophages 20 hours after activation with endotoxin. In vivo HMGB1 is detected in serum beginning 20–72 hours after the onset of the infection. HMGB1 appears to be necessary and sufficient mediator for the severe sepsis because systemic HMGB1 is found in patients and experimental animals with severe sepsis [10]. Administration of recombinant HMGB1 to mice recapitulates the characteristic organ dysfunction of severe sepsis, including derangement of intestinal barrier function, acute lung injury, and lethal multiple organ failure [11–15]. The inhibition of HMGB1 secretion or activity prevents endotoxin- or bacteremia-induced multiple organ failure [11]. Neutralizing antibody or “A-box”, a truncated DNA-binding domain, acts as a competitive inhibitor of HMGB1. Neutralizing antibody represents a therapeutic agent which makes HMGB1 an effective target to reverse the established sepsis in a clinically relevant time [11]. Ethyl pyruvate, an anti-oxidant that inhibits HMGB1 secretion from macrophages, can be added a day after TNF production to reverse the established sepsis [16, 17].

## 3. Pathophysiology of Sepsis

The reticuloendothelial system (RES), part of the immune system, consists of the phagocytes, monocytes, and macrophages accumulating in lymph nodes and the spleen. The Kupffer cells of the liver and histiocytes are also part of the RES. RES cells primarily target the pathogens or toxins in the process of sepsis in the lung, liver, and spleen. After infection, leukocytes are localized in sinusoids, perisinusoidal and perivascular spaces of the liver and lungs. In the spleen, leukocytes are localized in follicles of the red and white pulps gathering with hematopoietic cells. Spleen is a critical organ directing the innate immune responses and modulating inflammation. Spleen has two important immune functions: producing specific antibodies and serving as a filter system for opsonized bacteria in the blood stream. There are plenty of hematopoietic cells in the spleen, which are also involved in the inflammation. The growth factors, cytokines, and interferon-gamma all play an important role in proliferation and differentiation as well as apoptosis of these cells. IL-1, IL-6, and TNF released by activated monocytes and macrophages have been confirmed in vitro and in vivo to induce lymphocyte apoptosis and activate lymphoid cells [18, 19]. The inflammation associated functional incapacity of macrophages to release proinflammatory cytokines IL-1 and IL-6 inhibits the activity of caspase 1 and enhances the activities of caspase 3, 8, and 9, which are responsible for the endonuclease activation causing apoptosis [20]. 

**Figure 1:**
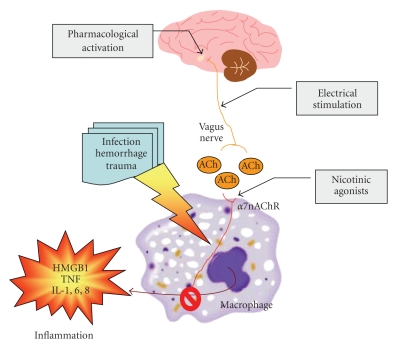
Nicotinic anti-inflammatory pathways. The inflammatory response to the infection, hemorrhage, or tissue trauma is mediated through the pathways of cytokine HMGB1, TNF, and interleukins which are released from immunized cells of lymph system including spleen tissue. Such immune pathway can be modulated by vagus nerve via its acetylcholine transmitter through either muscarinic or nicotinic receptor. A nicotinic acetylcholine receptor (*α*7nAChR) agonist, such as nicotine, or transmitter released from vagus nerve stimulation, can inhibit the inflammation through restraining the production of proinflammatory cytokines from lymphocyte macrophage line. As cholinergic selective, nicotine is more efficient than acetylcholine in anti-inflammatory response through *α*7nAChR-dependent mechanism.

The innate immune system protects against infection and tissue injury through the specialized organs of the RES, including the lungs, liver, and spleen. The central nervous system regulates innate immune responses via the vagus nerve; a mechanism termed the cholinergic anti-inflammatory pathway. Vagus nerve stimulation inhibits cytokine production by signaling through the alpha7nAChR [6]. Previously, the functional relationship between the cholinergic anti-inflammatory pathway and the RES was unknown. Now it has been shown that vagus nerve stimulation fails to inhibit TNF production in splenectomized animals during lethal endotoxemia [21]. Surgical sectioning of the common celiac nerve abolishes the TNF suppression by vagus nerve stimulation, suggesting that the cholinergic pathway is functionally connected to the spleen via this branch of the vagus nerve. Administration of alpha7nAChR agonist nicotine can mimic vagus nerve stimulation and inhibit release of proinflammatory cytokines from immunized cells of lymph system including spleen tissue. These results reveal a specific and physiological connection between the nervous and innate immune systems that may be exploited through either electrical vagus nerve stimulation or administration of alpha7nAChR agonists to inhibit proinflammatory cytokine production during infection and tissue injury (Figure 1).

Lung injury and a severe form called acute respiratory distress syndrome (ARDS) represent a major portion of mortality in intensive care units in the US [22]. An increase in HMGB1 levels were seen in plasma and epithelial lining fluids in lung injury [23]. Intratracheal administration of recombinant HMGB1 induced a dose-dependent interstitial and intra-alveolar neutrophil accumulation and lung edema at 8 and 24 hours postadministration [22]. There were increased levels of proinflammatory cytokines MIP-2, TNF, and IL-1 [24]. Accordingly, HMGB1 antibodies reduced this neutrophil accumulation, edema, and lung permeability. Instillation of endotoxin in mice caused inflammatory cell migration to lungs, destruction of pulmonary parenchyma cells, pulmonary hemorrhage, and acute lung injury [23]. High levels of HMGB1 were detected in plasma and bronchiolar fluid in LPS instilled mice, implicating an involvement of macrophage cells. Similar acute lung injury was identified in a polymicrobial peritonitis model of severe sepsis [24]. 

Bacteria translocate from the gut to systemic organs, but the route they take is unclear. Zymosan, which activates the complements, was used to induce a systemic inflammation in rats. At a lower dose (0.1 mg/g) the bacteria translocated to the mesenteric lymph node complex, whereas at a higher dose (0.5 mg/g) the bacteria translocated systematically via the portal vein rather than the mesenteric lymph [25]. As a first line of defense, macrophages become activated, and are specifically adapted to respond in an attempt to localize or ward off the foreign pathogens. Uncontrolled activation of macrophages and the loss of intestinal barrier function have been implicated in the development of ARDS and multiple organ failure. Influence of drug-induced macrophage elimination on zymosan-mediated systemic inflammatory responses was tested in a project study [26]. Macrophage-depleted mice were challenged for 2 days with different doses of zymosan (0.1 to 1.0 mg/g). 24 hours after the challenge, systemic stress was determined and translocation of bacteria to mesenteric lymph node, liver, spleen, and blood was measured. The macrophage-depleted mice had significant high-bacterial spreading systemically and low-toxic reaction of zymosan with the 12-day mortality rates 0% compared to 27% in the control animals [27]. Therefore, the toxic responses of animals to zymosan were highly associated with an excessive activation of macrophages rather than the systemic spread of bacteria.

Dendritic cells (DCs) that respond to foreign invasion and function as antigen-presenting to mature B cells to be specific antibody producing factories are also involved in systemic sepsis. Depletion or impaired function of DCs attributes to the mortality of the sepsis. To study the role of DC immune in LPS-induced sepsis, a transgenic mouse model with DCs overexpressing a human form of anti-apoptotic protein Bcl-2 (DC-hBcl-2 mice) has been used [28]. The transgenic mice DCs maintain the differentiation potential of Th1 cells to enhance T cell activation. Bcl-2-producing DCs are higher resistance to LPS-induced DC loss and immunosuppression, and improves animal survival in LPS-induced shock in DC-hBcl-2 mice. Therefore, DC death was proven as a key determinant of endotoxin-induced immunosuppression and mortality in mice.

## 4. Comparison of Experimental Models of Sepsis

The body may develop the inflammatory responses to microorganisms (or microbes) in the circulatory stream, urine, skin, or any organ tissues. On the contrary, systemic inflammatory response syndrome (SIRS) may occur in patients without the presence of infection, such as in those with trauma, burns, hemorrhage, or some nonspecific inflammatory diseases like chemical pneumonitis, pancreatitis, and so forth [29]. Sepsis may become severe when inflammatory response leads to organ dysfunction due to hypotension or septic shock. The organ dysfunction may occur in one or more organs, which is called multiple organ dysfunction syndrome [30]. From cell signaling pathway and molecular biological perspective [31], the balance between the pro- and anti-inflammatory cytokines is disturbed in SIRS. In addition to over production of TNF, IL-1, IL-2, IL-6, and IL-12, other inflammatory mediators such as C5a involve the complement cascade. The complement cascade associated mediators unbalance between the coagulation and fibrinolysis systems, and between the adrenergic and cholinergic pathways. The overwhelmed clotting system and neuron transmitters contribute to an immunosuppression or anergy. In order to study, this multitude of activity, which may eventually lead to beneficial clinical advance, experimental animal model systems which attempt to emulate systemic sepsis are devised. The most common are discussed below.

Endotoxemia refers to the presence of endotoxins in the blood. Endotoxemia emulates sepsis by intravenous administration of endotoxin lipopolysaccharide (LPS), a cell membrane component of Gram negative bacteria. Rodent models are the most common representatives, but they have small blood volume and are relatively resistant to endotoxin. A large dose of endotoxin results in sudden cardiovascular collapse and death [32]. Baboons more closely resemble human being in endotoxin sepsis with typical coagulopathy and progressive multiorgan dysfunctions [33]. The insult of LPS presumes that it is the host response, rather than intact pathogen that causes the clinical features of sepsis. LPS sepsis increases proinflammatory cytokines in serum with septic clinical manifestations, which are closely mimicked with administration of recombinant TNF or IL-1 to the animals [8, 34]. Mice injected with LPS peaked the cytokines at 3 hours and started to decline at 8 hours, whereas in the cecum ligation and puncture (CLP) model (described below) the cytokines had higher level at 8 hours and continued to increase thereafter. Although mortality rates were similar in both LPS and CLP models, the significant differences in the kinetics and magnitude of cytokine production suggested that the CLP model more accurately had the cytokine profile of sepsis. 

Sublethal and lethal doses of endotoxin or living E. coli in nonhuman primates resulted in a severe disseminated intravascular coagulation [35]. The inflammatory and hemostatic responses involved the microvascular endothelium and regulatory anticoagulant networks, which contributed to multiorgan dysfunction. Pretreatment with a TNF inhibitor had hemodynamic improvement and better survival in baboons, who otherwise exhibited an exaggerated TNF response with cardiovascular collapse and early death from a LD100 dose of E. coli [32]. Anti-TNF or its soluble receptor is protective in LPS animal models but doest not improve survival in clinical trials for most patients. LPS may be a good model for endotoxic shock, and can demonstrate some features such as early production of cytokines, but does not adequately resemble the pathophysiology typically to the evolving multiorgan dysfunction presented in the sepsis. 

CLP is another experimental model of sepsis to emulate human sepsis, but a more natural replica of peritonitis. Here the cecum is perforated, ligated, and the natural contents of the colon are leaked into the peritoneum, causing polymicrobial infection. The leakage sequentially results in bacteremia, sepsis, septic shock, SIRS, and eventual death. The cytokine profile is different from that of LPS infusion. In LPS, early and high transitory levels of IL-1, TNF, IL-6, and hypothermia or other clinical symptoms appear within a few hours. In CLP the levels of these proinflammatory cytokines and mortality go up proportionately to the length of ligated cecum [36]. IL-1, IL-6, and C5a reach moderately high level at 12–24 hours; and serum levels of LPS are minimum or even undetectable at this time similar to that in human sepsis. At this time point, anti-C5a or anti-IL-6 rather than anti-LPS or anti-TNF is more protective to the animals [37–39]. Although consistency of surgical procedures is a factor, CLP is currently a typical standard in experimental models of sepsis.

## 5. Systemic Inflammation in Hemorrhage

Severe hemorrhage is a common cause of morbidity and mortality in critical care medicine [40–42]. Hypovolemia and hypotension that limit the oxygen consumption and cause oxidative stress and organ injury determine the prognosis. After losing over ~50% of the total blood volume, the system becomes unable to reestablish tissue perfusion, causing lethal cardiovascular shock and multiple organ failure. During severe hemorrhage and fluid resuscitation, an overwhelming production of inflammatory cytokines such as TNF and HMGB1, and signs of SIRS, is usually present. Practically, the conventional resuscitation fluid is first used to reperfuse the tissues. However, this is unable to prevent inflammatory responses and the SIRS [41–43].

Hemorrhagic shock is conceived as a global ischemic insult to frequently cause SIRS with tissue damage and multiple-organ dysfunctions [44], which involves a complex mechanism in pathogenesis. The most widely recognized ways appear to be associated with ischemia, reperfusion, and stimulation of cells of the innate immune system. Ischemia and reperfusion is known to result in oxidative; and SIRS is found to be obvious during or postischemic resuscitation [45, 46]. The extent of tissue ischemia, that defines the degree of oxygen debt, correlates with a systemic inflammatory response that renders the injured patient at risk of postresuscitation multiple organ dysfunctions [47]. Regarding the duration of hemorrhagic shock it has been shown that the longer the shock persists the more intense is the inflammatory response that follows [48]. Similarly, survival seems to be improved with early resuscitation, and mortality to be high with delayed resuscitation [49]. It has been shown that the severity of shock is a more important determinant than the duration of shock in generating a higher mesenteric lymph flow at the postshock period. Likewise, this lymph engenders greater bioactivity as measured by human polymorphonuclears (PMN) priming for respiratory burst [50]. 

TNF is considered as pivotal inflammatory agent in hemorrhage. TNF diffuses in the bloodstream and initiates a fatal cardiovascular collapse [43]. TNF is a sufficient and necessary mediator of hemorrhagic shock, because (i) it is detected to be rich in patients and experimental models of hemorrhagic shock; (ii) it may contribute to the lethality of hemorrhagic shock; (iii) its neutralization attenuates cardiovascular shock. TNF plays an important role in excessive autodestructive inflammation and may finally induce multiple organ failure through the activation of neutrophils [51]. TNF is known to trigger the release of other proinflammatory cytokines, such as IL-1b and IL-6 [52]. In addition, TNF has much more potency than that of other cytokines in activation of neutrophils [53]. Many experimental studies have focused on the production of TNF in severe hemorrhage (loss of 50% of the total blood volume) or prolonged hemorrhage with fluid resuscitation. Recently, it has been shown that ethyl pyruvate can inhibit TNF production from human and murine macrophages [16, 17]. In vivo, ethyl pyruvate attenuated systemic inflammation and improved survival in experimental models of hemorrhage [54–56].

**Figure 2:**
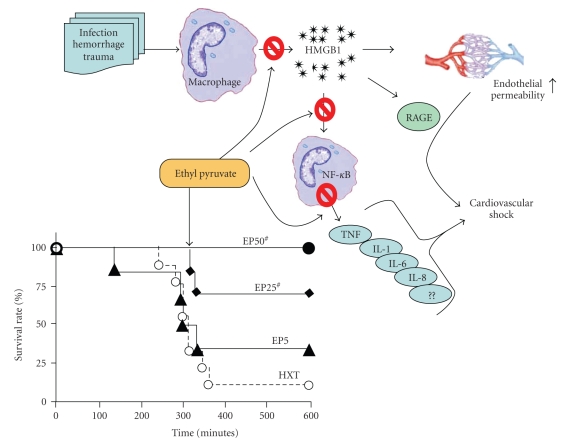
Inflammatory response of HMGB1 and its attenuation by ethyl pyruvate. HMGB1 is liberated from macrophages into the extracellular milieu following cell membrane perturbations during sepses, hemorrhage, and injury. HMGB1-mediated nitric oxide (NO) leakages the endothelial line leading to a high permeability. NFKB signally stimulated by HMGB1 in macrophage regulates the release of TNF cytokine and interleukins. Inflammatory shock and animal death result due to vascular collapse and cytokine toxicity of the organ tissues. By attenuating the HMGB1 pathways with hextend-ethyl pyruvate (HEP), animals may improve survival with the dependent dosage.

In addition to TNF, recent studies indicated that HMGB1 also plays an important role in hemorrhagic shock. HMGB1 was originally identified as a nuclear DNA-binding protein that functions as a structural cofactor. However, during cellular damage or necrosis, HMGB1 is liberated into the extracellular milieu where it functions as an inflammatory cytokine [57–59]. Extracellular HMGB1 sustains inflammatory responses producing abrupt cardiac standstill, intestinal derangement and acute lung injury [9, 60–62]. For these reasons, there is a major interest in inhibiting the production of these factors during resuscitation (Figure 2).

## 6. Comparison of Experimental Models of Hemorrhage

Hemorrhage is frequently associated with trauma. The immune response to hemorrhage would be expected to be different in conscious versus unconscious individuals; and anesthesia and analgesics have different immune consequences; hence the different physiological and immune responses may occur and different treatment regimens may need to be applied. For example, Phenobarbital increases extra-alveolar permeability and promotes neutrophil recruitment in the lungs [63]. Morphine induces immunosuppressive effects mediated by the induction of corticosterone [64]. Many animal models have been used for trauma/hemorrhage research [65]. For these reasons, we studied hemorrhage and resuscitation in experimental rat models of conscious (without anesthesia), anesthetized, and anesthetized plus trauma. Rat hemorrhage was normalized by constant pressure rather than constant volume in order to directly compare the 3 conditions. Rats were bled to a mean arterial pressure (MAP) of 35–40 mmHg. Many different bleeding protocols have been used to emulate real life conditions [51]; and a recent study claimed to emulate true conditions by varying rate of blood loss that maximized physiologic insult [66]. In our studies, the conscious rats took more shed blood, and the anesthetized ones with trauma took less blood than the traditional anesthetized model to achieve the similar MAP result. Differences in shed blood did not correlate with survival. The nonresuscitated rats of anesthetized and anesthetized plus trauma had a similar average survival time of 160 and 167 minutes, respectively, whereas the conscious rats had a statistically significant shorter average survival time of 87 minutes in spite of their ability to vasoconstrict and compensate blood loss, which was neutralized by the constant pressure regimen [67]. 

Advanced resuscitation fluid, Hextend, a novel plasma volume expander containing 6% hydroxyethyl starch in Ringer's lactate was used for resuscitation, which significantly improved survival in all models [67]. Hetastarch creates oncotic pressure, which is normally provided by blood proteins and permits retention of intravascular fluid. Although Hextend protected the animals of anesthetized plus trauma the most in survival rate, 40% (*P* > .01 versus nonresuscitated) as compared to 25% for conscious and 10% for the anesthetized only, it failed to increase the average survival time (delay of death after hemorrhage) in animals with anesthesia and trauma as compared to the other two models. All nonsurvival animals died within 7 hours of hemorrhage, and all others survived for 5 day follow-up period. Blood chemistries were measured at 2 hours after hemorrhage, which was the average time of death for nonresuscitated conscious animals. All models had similar uremia, which was not corrected and prevented by Hextend resuscitation. All models had acidosis (increased Anion Gap) with conscious model being significantly higher than the others; however, Hextend resuscitation significantly restored AnGap back to normal level in conscious model. All models had hyperglycemia. Serum TNF levels measured (by ELISA) at that time demonstrated 2-fold significantly higher (*P* < .05) in rats of conscious and anesthetized plus trauma than animals with anesthesia. TNF levels measured in buffer-washed heart also had parallel 2-fold significant increase (*P* < .01) in anesthetized plus trauma and conscious rats over anesthetized animals. All washed organs taken for analyses had TNF values 3 orders of magnitude higher than serum levels, (i.e., organs/serum = *μ*g/pg) expressed by protein-standardized amounts. The lung and liver had significantly higher TNF levels in all models with the highest in conscious rats. The spleen showed similar results in anesthetized plus trauma model, but was not as clear for the others [67].

## 7. Pathophysiology of Hemorrhage

It is speculated that the spleen is a major source of systemic TNF content in hemorrhage. Inhibition of TNF production in the spleen prevents cardiovascular shock [6, 68]. In a rat model of nonlethal hemorrhagic shock, TNF carried in the lymph or portal vein would promote the endothelial cell damage and activate the neutrophils to a greater extent [69]. The samples were collected from efferent lymph duct draining the mesenteric lymph node complex 6 hours after sublethal hemorrhagic shock. As well, the portal vein plasma was collected, and both samples were tested for endothelial cell (HUVEC) monolayer permeability. The lymph but not portal vein plasma from postshock was showed to increase the HUVEC permeability and to be cytotoxic to endothelial cells. Postshock lymph induced a greater formation in neutrophil superoxide and deteriorated the neutrophil-mediated endothelial cell damage during incubation. 

Since the lung is the first organ coming in contact with mesenteric lymph, its injury due to hemorrhagic shock might be caused by factors present in mesenteric lymph as compared to the portal vein plasma. An outbreak of lung dysfunction may be directly related to the release of TNF and IL-1 beta during acute hemorrhage [70]. Except for spleen and lymph system, heart is also shown to play an important role in the expression of TNF and IL-1 [71–73]. These cytokines diminish cardiac contraction by direct parenchymal damage with calcium handling disruption [72, 74]. In addition, they are important mediators of induction of leukocyte chemotaxis in conjunction with adhesion molecules such as beta-4 integrin, intercellular adhesion molecule 1, and neutrophils. An outbreak of fatal heart dysfunction or failure caused by hemorrhagic shock was reported [70, 75, 76]. The p38 mitogen-activated protein kinase (p38 MAPK) is activated in the cardiac ischemic state [77–79], which promotes cellular stress responses such as proliferation, differentiation, and production of the proinflammatory cytokines [77, 80, 81]. Several investigators demonstrated that p38 MAPK in the cardiac ischemic state was activated not only in macrophage or endothelial cells but also in cardiac myocytes [77, 81], and that the activation occurred immediately after the ischemic induction [15]. The beta-2 integrin and vascular-cell adhesion molecule 1, may involved in activation [82–84]. The adhered neutrophils are known to generate and release numerous active substances such as proteolytic enzymes and reactive oxygen species. All of them have the potential to damage the endothelial layer and adjacent tissue [70, 80, 85]. The activation of p38 MAPK caused myocardial apoptosis, resulting in cardiac dysfunction within a short time after myocardial infarction [86].

## 8. Comparison of Systemic Inflammation in Sepsis and Hemorrhage

The balance of pro- and anti-inflammatory mediators derived from the innate immune system defines the progression and severity of sepsis and hemorrhage. An overproduction of endogenous proinflammatory mediators, including cytokines, platelet-activating factor, oxygen radicals, and nitric oxide, synergistically interact to mediate hypotension, multiple organ failure, and death. Progression from sepsis to septic shock coincides with the increase in circulating levels of proinflammatory cytokines such as TNF, interferon gamma, IL-1, and IL-6 [87]. Studies from animal models suggest that inhibition of these early mediators suppresses particular facets of the pathological response. For instance, neutralizing antibodies to TNF, the first cytokine elaborated in the septic inflammatory cascade, prevent death when administered before or concurrent with lethal doses of live Escherichia coli [9]. TNF is a necessary and sufficient mediator of septic shock in experimental animal models. First, TNF is produced in animals during septic shock. Second, removing TNF from diseased animals, by either pharmacological strategies or genetic disruption, significantly improves survival after endotoxin challenge. Last, administration of TNF to normal animals reproduces the pathological sequelae of septic shock [9, 88, 89]. The identification of TNF as an essential mediator of Gram-negative septic shock focused attention on the development of therapies directed at endogenous toxins. A number of other proinflammatory cytokines have since been implicated in the mediation of endotoxin lethality. For instance, IL-1 [90, 91], leukemia inhibitory factor (LIF) [92, 93], IFN gamma, and migration inhibitory factor (MIF) may each contribute to the pathogenesis of endotoxemia or septic shock. Clearly, the pathogenesis of sepsis is modulated by an interaction between these and perhaps other mediators. Each of these secondary mediators can be induced by TNF. 

The early systemic release of TNF during endotoxemia activates lethal downstream pathological responses [91]. Endotoxin also initiates anti-inflammatory mechanisms that down regulate or suppress potentially injurious proinflammatory mediators. In LPS-stimulated monocytes, for example, the local accumulation of prostaglandin E2 (PGE2) inhibits the synthesis of proinflammatory cytokines that can restrain an acute cytokine response [94]. Anti-inflammatory cytokines (e.g., IL-10 and transforming growth factor beta [TGF-beta]) are involved in the downregulation of the inflammatory response. For instance, IL-10 can deactivate macrophages; and the TNF levels in patients with trauma get higher when IL-10 levels are depressed, a scenario that has been implicated in the onset of septic complications [95]. TGF beta is a potent inhibitor of monocyte activation [96]; and significantly elevated levels of TGF beta have been observed in monocytes derived from immunosuppressed patients after trauma. New evidence suggests that the central nervous system can directly, and rapidly, attenuate the TNF response to endotoxin through efferent vagus nerve signals to tissue-resident macrophages [97]. This effect is mediated by acetylcholine, the principal neurotransmitter of the vagus nerve, which signals via nicotinic cholinergic receptors present on macrophages [98]. Thus, the complex cytokine milieu in septic patients is characterized by the interaction between anti-inflammatory responses and potentially injurious proinflammatory responses that are tightly regulated by neural and humoral pathways. 

Hemorrhagic shock is a severe life-threatening emergency affecting all organ systems of the body by depriving tissue of sufficient oxygen and nutrients owning to decreased cardiac output. Moreover, resuscitation can trigger systemic inflammatory responses that can be more dangerous than the initial hemorrhage. Conventional resuscitation fluids are designed to restore circulatory volume and tissue perfusion, but they failed to prevent inflammatory responses. An acute loss of blood results in a cascade of compensatory events that affect all organ systems. The initial response to hypovolemia is to decrease circulation to less vital organs such as the kidneys, gut, and skin in order to preserve circulation to priority organs such as the heart, brain, lungs, and skeletal muscle. This shunting to vital organs is triggered from a decrease in cardiac output and subsequently pulse pressure, which is sensed by baroreceptors within the aortic arch and atrium. Neural reflexes then cause a sympathetic outflow to the heart and other organs, which respond by increasing heart rate and vasoconstriction. A hormonal response occurs, and activation of the renin system leads to vasoconstriction and the retention of sodium and water. The anterior pituitary and adrenal medulla are stimulated to release adrenocorticotropic hormone, epinephrine, and norepinephrine, which enhance compensatory mechanisms. At the cellular level, a decrease in perfusion causes the cells to switch from aerobic to anaerobic metabolism. Lactic acid is formed and that causes metabolic acidosis. If the blood loss continues, the compensatory mechanisms begin to fail and damage occurs throughout the body [99, 100].

## 9. Summary

Both sepsis and hemorrhage can develop lethal inflammatory responses or systemic inflammatory response syndrome (SIRS). Sepsis can represent an extrinsic activation in response to infection or bacterial endotoxins. Hemorrhage can activate similar responses though an intrinsic response to tissue damage. In both cases, overwhelming inflammatory responses can become more dangerous than the original pathogenic insult. In this case, efficient anti-inflammatory strategies can provide therapeutic benefits for both septic and hemorrhagic clinical settings.
